# Biosynthesis of palladium, platinum, and their bimetallic nanoparticles using rosemary and ginseng herbal plants: evaluation of anticancer activity

**DOI:** 10.1038/s41598-024-56275-z

**Published:** 2024-03-09

**Authors:** Moloud Alinaghi, Pooneh Mokarram, Mazaher Ahmadi, Farzaneh Bozorg-ghalati

**Affiliations:** 1 Autophagy Research Center, Department of Biochemistry, School of Medicine, Shiraz University of Medical Sciences, Shiraz, Iran; 2 Faculty of Chemistry and Petroleum Sciences, Bu-Ali Sina University, Hamedan, Iran

**Keywords:** Palladium nanoparticles, Platinum nanoparticles, Green syntheses, Rosemary, Ginseng, Anticancer, Biochemistry, Cancer, Molecular biology

## Abstract

In this research, palladium (II) and platinum (II), as well as their bimetallic nanoparticles were synthesized using medicinal plants in an eco-friendly manner. Rosemary and Ginseng extracts were chosen due to their promising anticancer potential. The synthesized nanoparticles underwent characterization through FT-IR spectroscopy, DLS, XRD, EDX, SEM, and TEM techniques. Once the expected structures were confirmed, the performance of these nanoparticles, which exhibited an optimal size, was evaluated as potential anticancer agents through in vitro method on colon cancer cell lines (Ls180, SW480). MTT assay studies showed that the synthesized nanoparticles induced cell death. Moreover, real-time PCR was employed to investigate autophagy markers and the effect of nanoparticles on the apoptosis process, demonstrating a significant effect of the synthesized compounds in this regard.

## Introduction

In recent times, substantial research efforts have been dedicated to the synthesis of various metal nanoparticles, including but not limited to silver, palladium, platinum, gold, iron, zinc using environmentally friendly methods. These nanoparticles have been explored for their catalytic and biological applications^1–4^. Over the past decade, there has been a noticeable shift towards green synthesis methods, involving the identification and optimization of these methods, along with investigating the structure and characteristics of synthesized nanoparticles^5–7^. In more recent studies, particular emphasis has been placed on the practical applications of synthesized nanoparticles across various domains^8–12^. For example, nanoparticles prepared using different microorganisms, including bacteria, algae, yeast, and fungi, and extracts of various plant components (leaf, root, stem, fruit) have shown various applications in both catalysis and bio-medical field^13,14^. Researches in recent years show that metal and metal oxide nanoparticles prepared using green methods have shown important potential environmental and biomedical applications^15–20^. Among various metallic nanoparticles, the synthesis of platinum and palladium-based nanoparticles via various methods has been at the center of attention, primarily owing to their outstanding usages the field of medicine. Throughout history, platinum-based medications, notably cisplatin and carboplatin, have been employed in cancer chemotherapy. More recently, platinum nanoparticles (PtNPs) have emerged as a subject of interest. Additionally, palladium nanoparticles (PdNPs) have demonstrated potential for combating leukemia and breast cancer. While various physical and chemical synthesis methods exist for these nanoparticles, biological approaches are favored for their superior biocompatibility and non-toxic nature, which is essential for effective nanomedicine^21–24^. Palladium and platinum nanoparticles, synthesized through diverse methods have exhibited notable antimicrobial and anticancer properties^25–27^. Numerous studies have proven their role in inducing apoptosis in cancer cells^28–30^. Additionally, the therapeutic potential of medicinal plant extracts in cancer treatment has been the subject of extensive investigation^31–34^. So far, considerable research has been conducted in the field of biological investigation and pharmacological effects of medicinal plant extracts, with particular emphasis on the plants employed in this study, namely rosemary and ginseng. Several reports indicate the diverse range of therapeutic effects offered by extracts from these plants, especially in the context of disease treatment, notably various forms of cancer.

Rosmarinus officinalis, also called rosemary, is a perennial, evergreen shrub of the Lamiaceae family^35^. Originally native to the Mediterranean region, rosemary is now grown worldwide, primarily for its role as a natural food preservative and flavor enhancer^36^. This herb has a rich history in traditional medicine for centuries, with uses encompassing memory improvement and the controlling digestive ailments^37,38^. The efficacy of rosemary in preserving and healing can be attributed to its essential oil and extract. Notably, phenolic compounds like carnosic acid, carnosol, and rosmarinic acid are responsive for the potent antioxidant properties found in rosemary's essential oil and extract^39^. These remarkable antioxidants not only contribute to rosemary's effectiveness as a natural food preservative but also underlie its diverse therapeutic applications, including its potential against cancer and diabetes^40^. Consequently, the robust antioxidant components present in rosemary essential oil and extract have elevated this plant to a prominent position within the food and medical industries. It should be noted that the European Food Safety Authority (EFSA) has acknowledged this extract as a beneficial compound. Remarkably, rosemary exhibits hepatoprotective, anti-spasmodic, anti-cancer, anti-tumor, anti-microbial, anti-diabetic, anti-inflammatory, and antioxidant attributes^41,42^. These inherent biological attributes position rosemary as a promising prospective therapeutic agent e curing numerous ailments. Numerous investigations have delved into the mechanisms by which this plant combats cancer. Rosemary exhibits dramatic antiproliferative effects facing diverse human cancer cell lines. Primary compounds in rosemary extract, including carnosic acid, carnosol, and rosmarinic acid, are believed to trigger apoptosis in cancer cells, likely through the generation of nitric oxide, with carnosic acid being the most potent inducer^43,44^. Furthermore, rosemary extract demonstrates noteworthy anti-tumor properties^45,46^. The anti-tumor influence of rosemary is pertinent to a variety of mechanisms, encompassing antioxidant actions, anti-angiogenic characteristics, epigenetic effects, modulation of immune and anti-inflammatory responses, adjustments in specific metabolic pathways, and upregulation of suppressive genes^47–50^.

The second botanical extract incorporated into this study is derived from ginseng roots. Ginseng comprises several plant species, with over a dozen of the *Panax* genus within the Araliaceae family. With a rich heritage in traditional Chinese medicine (TCM), ginseng is renowned for its diverse array of bioactive compounds, encompassing ginseng saponins, fatty acids, polysaccharides, and mineral oils. Within ginseng, one of the noteworthy bioactive compounds is ginseng saponins, a category of natural steroidal glycosides and triterpene saponins unique to this plant. Extensive research has emphasized the pivotal role of these ginseng saponins in conferring a wide range of therapeutic benefits. Literature reviews delve into their chemical structure, categorization, and explore their diverse pharmacological activities, encompassing their influence on immune regulation, anticancer properties, as well as their impact on the central nervous and cardiovascular systems. Furthermore, these saponins are associated with enhancing physical stamina and overall quality of life. Additionally, valuable recommendations for further research on the utilization of ginseng saponins as robust therapeutic agents have been documented^51^. Lately, research has been conducted in various fields of medical applications of the ginseng plant, including regulatory effects on the immune system, anticancer effects, and its impacts on both the cardiovascular and central nervous system^51^. The primary composition of ginseng saponins consists of a hydrophobic, steroidal four-ring structure^52,53^. These compounds have been associated with enhancing the overall immune system, reducing inflammation, protecting against hepatotoxicity, and exhibiting safeguarding effects against various mammalian tumor cell lines and nonspecific cancers. Moreover, ginseng saponins have demonstrated a range of pharmacological effects, encompassing cardiovascular protection, anti-inflammatory properties, antiviral actions, and immune system regulation, often employing novel mechanisms^54^. Ginseng saponins are well-recognized for their notable anti-tumor characteristics, primarily attributed to their capacity to reduce inflammation, inhibit cell proliferation, impede metastasis, and counteract angiogenesis. Furthermore, their minimal toxicity and limited adverse effects introduce them as a promising approach for further exploration in anticancer research. Ginseng saponins exhibit anticarcinogenic properties both in laboratory settings and within living organisms through diverse mechanisms, such as direct cell toxicity, growth inhibition, stimulation of differentiation, and the prevention of metastasis^55–58^.

The mechanism which lies behind the creation of metal nanoparticles from plant extracts has been mentioned in earlier studies^59–61^. In all instances, it has been observed that metal salt reduction reactions are initiated by various natural reducing agents found within plant extracts. These agents include flavonoids, proteins, enzymes, polysaccharides, alkaloids, terpenoids, aldehydes, carboxylic acids, and so forth. Basically, the formation of nanoparticles by this method consists of two important steps: first, nucleation and then particle growth in the second step, and it requires the reduction and stabilization of mentioned organic molecules^62–65^. What sets plant chemical compounds apart is their ability to expedite the reduction of metal ions in significantly less time in comparison with fungi and bacteria, which require lengthier incubation periods. Hence, plant extracts have gained recognition as a safe and highly efficient source for the synthesis of both metal nanoparticles and metal oxides. Moreover, plant extracts serve a dual purpose in the synthesis process, functioning as agents of reduction and stabilization, thereby facilitating the generation of nanoparticles. It's worth noting that several factors come into play when synthesizing nanoparticles through this method, influencing their size and shape^62,66–68^.

Prior studies have shown the efficacy of the plants used in this research for synthesizing nanoparticles with applications in the biological and medical purposes. For example, a recent study delved into the synthesis of palladium nanoparticles through a rosemary plant extract, revealing acceptable antimicrobial and antifungal properties in resulting nanoparticles^69^. Additionally, there are reports indicating that iron nanoparticles (FeNPs), synthesized using rosemary plant extract, exhibit anticancer performance opposing various cancer cell lines. Hence, the rosemary-FeNPs exhibited an average size of approximately 100 nm and a PDI lower than 0.12, signifying a consistent nanoparticle size distribution. To evaluate cytotoxicity, the MTT cytotoxicity assay was employed on 4T1 and C26 cancer cell lines using both rosemary-FeNPs and rosemary extract. The findings indicated that rosemary-FeNPs demonstrated greater cytotoxicity compared to the whole extract when exposed to both cancer cell lines^70^. Also, several researchers have noted the effect of ginseng nanoparticles and metal nanoparticles synthesized with ginseng plant extract as anticancer and antimicrobial agents^71–74^.

Current study presents the synthesis of palladium and platinum nanoparticles by means of rosemary and ginseng medicinal plants. Palladium and platinum nanoparticles hold significant potential in the realm of medical science, particularly in areas such as cancer treatment, drug delivery, and infectious diseases. Therefore, the primary objectives of this project are to biosynthesize these nanoparticles using an environmentally friendly solvent (water), characterize their structures through various techniques, and explore their applications within the medical field. Numerous metal nanoparticles have been successfully prepared and validated using biological methods, establishing their anticancer properties and effectiveness as drug carriers. However, a comprehensive investigation into palladium and platinum nanoparticles synthesized from extracts of two specific plants, rosemary and ginseng, and their anticancer properties, is still lacking in the literature. To address this gap, the current research endeavors to fulfill this critical research goal.

## Materials and methods

### Reagents and apparatus

The study on plants complies with local and national guidelines and regulations. Rosmarinus officinalis leaves were sourced from the natural environment of Isfahan, Iran, while Red ginseng roots were obtained from an herbal store. The precursor chemicals, palladium (II) chloride (PdCl_2_), and hexachloroplatinic acid hexahydrate (H_2_PtCl_6_·6H_2_O), as well as polyvinylpyrrolidone (PVP), were provided from Sigma-Aldrich, USA. Analytical grade HCl and sodium hydroxide (NaOH) were obtained from Merck Specialities Pvt Ltd, India. Cell culture-grade DMSO, MTT (dimethylthiazolyltetrazolium bromide), ethidium bromide, and phosphate buffer saline (PBS) were supplied from Sigma-Aldrich Co. in Canada (Oakville, ON). Beclin-1, LC3-II, and p62 were acquired from Cell Signaling Technology Co. in the USA (Beverly, MA). GAPDH was supplied from Santa Cruz Biotechnology Inc. (Dallas, Texas, USA).

DLS Nano Particle Analyzer (SZ-100) was employed to find the size of nanoparticles. FT-IR analysis was done with FT/IR-6300, JASCO. X-ray diffraction (XRD) was conducted by XRD D8 ADVANCE, Bruker. EDX-MAP, SEM, and TEM analysis were done by EDAS TESCAN, MIRA II, Leo 1430 VP, and TEM CM120, respectively.

### Preparation of rosemary and ginseng extracts

To create rosemary plant extract, in the first step, the process began by thoroughly washing the needle-shaped leaves with distilled water and allowing them to air-dry at room temperature. Once completely dried, were fragmented into smaller pieces, and a fine, consistent powder was produced through grinding. In the following, 10 g of this plant powder were mixed with 100 mL of deionized water and heated to boiling (100 °C) over a 15-min duration. Then, the reaction container was left to gradually cool. The resulting solution was subsequently filtered through the use of Whatman No. 1 filter paper and maintained at 4 °C, ready for utilization in the subsequent phases of the experiments.

The procedure for preparing the red ginseng root extract closely resembled the previously described method. To begin, 25 g of ginseng root were rinsed a couple of times by distilled water and dried. Following this, it was divided into smaller pieces and completely smoothed and uniformed using a mortar. Subsequently, the ginseng mixture was subjected to boiling in 100 mL of deionized water for a duration of 30 min. After the cooling phase, similar to the previous method, the plant extract was filtered using Whatman filter paper, and the resulting solution underwent centrifugation at 10,000 rpm for 10 min to separate other suspended compounds from the primary extract. The resulting solution was then diluted to 100 mL using deionized water and stored at 4 °C as a stock solution for future experimentation.

### Synthesis of palladium nanoparticles

To achieve this, the process began with the preparation of a 10 mM aqueous solution of PdCl_2_. Then, 5 mL of rosemary extract was introduced into 95 mL of the metal ion solution. Following that, 100 µL of concentrated hydrochloride were added to the solution to convert PdCl_2_ into H_2_PdCl_4_·nH_2_O. Then, 1.11 g of PVP was dissolved in methanol and incorporated into the reaction mixture to enhance solubility and stability in aqueous medium. A 20 mL of methanolic NaOH solution was introduced drop by drop into the reaction mixture, and the solution was refluxed for 5 h at 100 °C. At the end, the resulting solution was put in the refrigerator, and after the desired period, sedimentation and solution stability were assessed. It was determined that the use of PVP yielded homogenous nanoparticles to an acceptable level, with no precipitation observed even after six months. Synthesis of palladium nanoparticles using ginseng extract was also done according to the same method except for the extract type.

### Synthesis of platinum nanoparticles

A 10 mM aqueous solution was prepared using H_2_PtCl_6_·6H_2_O. Subsequently, 5 mL of rosemary extract was inserted into 95 mL of metal solution. Following this step, 1.11 g of PVP were dissolved in methanol and added to the reaction mixture to enhance solubility and stability in aqueous medium. The reaction mixture is refluxed for 5 h at 100 °C. Finally, the resulting solution was refrigerated and after the specified timeframe, the level of sedimentation and solution stability were assessed. It was observed that the use of PVP yielded homogeneous nanoparticles to a satisfactory extent. The synthesis of platinum nanoparticles using ginseng extract followed the same procedure, differing only in the extended reaction time, which lasted for 6 h.

In all reactions, the determination of reaction completion was based on referenced articles and the observation of a shift in color from yellow to brownish-black^69,75–77^.

### Synthesis of bimetallic nanoparticles

To this end, different ratios (1:1, 1:2, and 2:1) of palladium and platinum metal ions were investigated. Following the method employed for monometallic nanoparticle synthesis, a 10 mM solution containing both metals was prepared. First, the palladium solution was introduced into the reaction container, and 100 µL of concentrated hydrochloride was added. Subsequently, the platinum solution was incorporated, followed by stirring vigorously. In this phase, 5 mL of plant extract was inserted, followed by the addition of 1.11 g of PVP, which was dissolved before introducing 20 mL of methanolic NaOH solution drop by drop into the reaction medium. The reaction mixture was refluxed for 3 h at 100 °C. All these steps were repeated for Pd/Pt concentration ratio of 1:2 and 2:1. Three reactions were performed using rosemary extract and three reactions were performed using ginseng extract. Finally, six blackish-brown solutions were obtained.

### Cell lines, culture, and treatment

Human colorectal cancer cell lines, specifically SW480 and LS180, were purchased from Bonyakhteh Company located in Tehran, Iran. We conducted experiments using cells within passages 3–6, obtaining fresh and validated cell lines from the same source after 6 passages. These cells were nurtured in Dulbecco’s Modified Eagle’s Medium high in glucose and glutamine (DMEM) provided by Bio Idea in Tehran, Iran. The culture medium was further enriched with 10% Fetal Bovine Serum (FBS) (Gibco™; Cat #: 16000044) and 1% penicillin–streptomycin (Gibco, (Waltman, MA, USA). They were stored in a humidified incubator with an atmosphere consisting of 95% air and 5% CO_2_, all at a temperature of 37 °C.

### MTT assay

The practicability of cells under varying empirical conditions was assessed using the MTT assay, which quantified the growth inhibitory impact of the compounds on cancer cell lines. This was achieved through the application of the 3-(4,5-dimethylthiazol-2-yl)-2,5-diphenyltetrazolium bromide (MTT) assay^78^. A known concentration of tumor cells introduced into the wells of a 96-well plate, and various compound concentrations (ranging from 0 to 1000 µmol dm^−3^) were applied. The plate was then incubated at 37 °C with 5% CO_2_ and 95% humidity for a duration of 24–48 h. Subsequently, 10 µL of MTT solution (5 mg/mL) was introduced into each well, thereafter followed by an additional 4-h incubation at 37 °C. The formazan produced, which was insoluble, was eventually dissolved by the addition of dimethyl sulfoxide (DMSO) at a volume of 100 µL per well. Following the complete dissolution of the dye, we measured the optical density (OD) at 570 nm using a reference wavelength of 630 nm using an enzyme-linked immunosorbent assay (ELISA) reader (Bio-Tek’s ELx808, USA). To determine the percentage of cell inhibition resulting from various treatments, we used the following formula: % Inhibition = 100 − [(test OD/non-treated OD) × 100]. It's important to note that in all experiments, the non-treated cultures consisted solely of DMSO solvent at a concentration equivalent to that in the test wells. We constructed an inhibition percentage graph across different concentrations to establish the values for the half-maximal inhibitory concentration (IC_50_)^79,80^.

### RNA extraction and real-time PCR (RT-PCR) analysis

The quantitative reverse-transcriptase polymerase chain reaction (q RT-PCR) technique was exploited to assess the relative expression levels of Beclin-1, LC3, and P62. Total RNA was extracted from sub-confluent cell cultures, in line with the manufacturer’s guidelines. Subsequently, cDNA synthesis was carried out using the cDNA synthesis Kit from Yekta Tajhiz, Iran. Primer sequences (as detailed in Table 1) were designed with the assistance of AlleleID software (version 7.73). All samples were scrutinized in duplicate, and a no-template control (NTC) was included using sterile nuclease-free water (ddH_2_O). The Q RT-PCR was executed using the QuantStudio™ 3 Real-Time PCR System (ABI Applied Biosystems) and SYBR Green Amplicon from Yekta Tajhiz, Iran. The relative expression levels of the target mRNAs were standardized to the internal standard, GAPDH expression levels. In the end, the relative quantification was conducted using the Applied QuantStudio™ Design and Analysis Software.

**Table 1:** The primer sequences employed in this study.

Gene	Forward primer	Reverse primer
Beclin-1	AGCTGCCGTTATACTGTTCTG	ACTGCCTCCTGTGTCTTCAATCTT
LC3	CGGTACAAGGGTGAGAAGCA	GAGCTGTAAGCGCCTCCTAAT
P62	AATCAGCTTCTGGTCCATCG	TTCTTTTCCCTCCGTGCTC
GAPDH	CGACCACTTTGTCAAGCTCA	AGGGGTCTACATGGCAACTG

## Result and discussion

### Synthesis and characterization of nanoparticles

**Figure 1:**
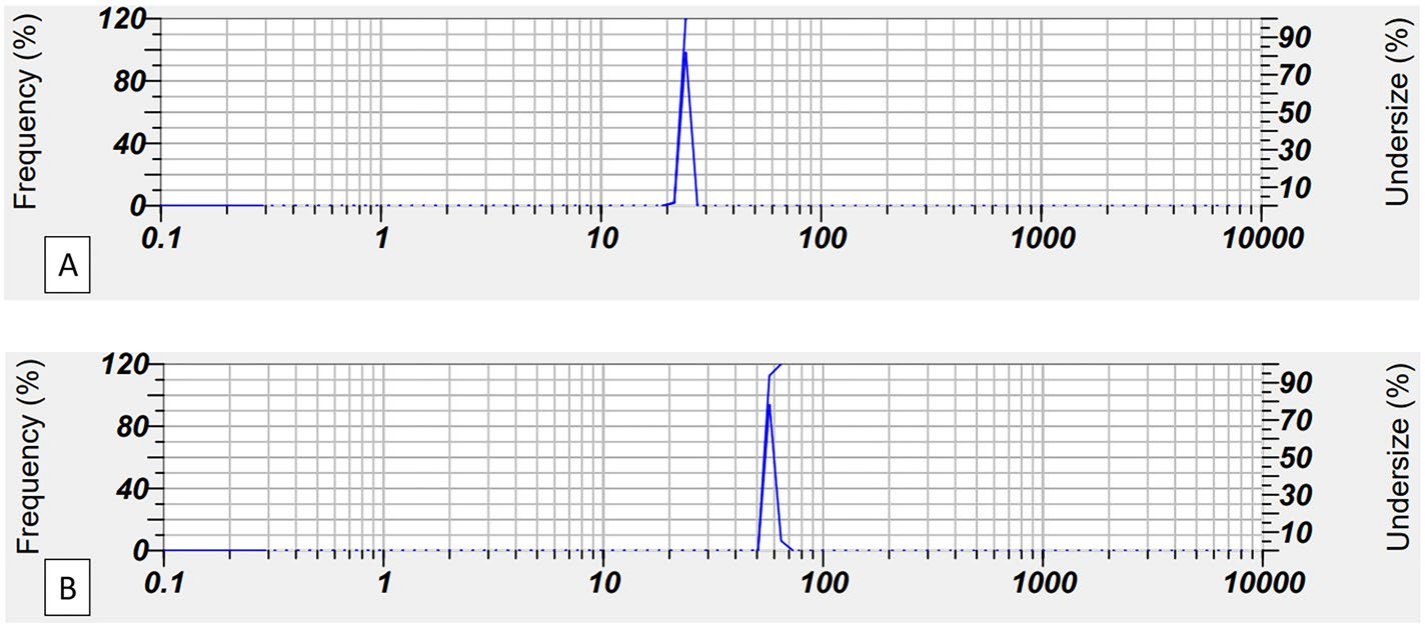
DLS analysis for 7 NPs (**A**) and 10 NPs (**B**).

The freshly prepared nanoparticles solutions underwent immediate dynamic light scattering (DLS) analysis. The resulting nanoparticle sizes can be found in Table 2. The findings revealed that employing various concentrations of metal salt solution in the synthesis reaction led to the formation of nanoparticles of varying sizes. Considering that the concentration of the metal solution affects the reaction speed, it also affects the size of the produced nanoparticles. Of course, other factors such as the nature of metal ions, the nature and concentration of the plant solution, the ratio of plant solution to metal solution, pH, temperature, and reaction duration, exerted an influence on nanoparticle shape and size^81^. For the synthesis of monometallic nanoparticles, the same concentration of metal solution was used to investigate the effect of the nature of the metal ions and the plant extracts. On the other hand, during the synthesis of bimetallic nanoparticles by changing the ratio of metals, it was observed that the nature of the metal has a significant effect on the size of the nanoparticles. The particle size analysis confirmed that all particles are synthesized in the nanometer scale. Smaller particles were formed in slower reactions, indicating that the rate of nanoparticle formation directly impacted their size. For example, platinum reactions, which were slower than palladium reactions, led to the synthesis of generally smaller nanoparticles. Also, in bimetallic nanoparticles, an increased ratio of palladium to platinum resulted in larger particles. The reason why platinum reactions are slower than palladium reactions can be explained by accounting the position of these two elements in the periodic table. On the other hand, reactions involving rosemary extract occurred at a faster rate than those using ginseng extract, leading to the production of larger nanoparticles. Note that the compounds existing in the plant extract influenced both the mechanism and the speed of the reaction^69^. Considering the size of nanoparticles, two bimetallic nanoparticles **7** and **10** were selected for future studies and investigations. The DLS analysis for nanoparticles 7 and 10 shows that they have sizes of 22.2 nm (PDI: 0.90) and 54.1 nm (PDI: 0.53), respectively (Fig. 1).

**Table 2:** DLS size of the synthesized nanoparticles. It should be noted that Rz, Gn, Pd, and Pt are abbreviations of Rosemary, Ginseng, Palladium, and Platinum, respectively. Also, the numbers indicate the concentration ratio of metal solutions during synthesis. Significant values are in bold.

Nanoparticles	Size (nm)	SD (nm)
1. Pd (Rz)	197.2	133.4
2. Pd (Gn)	201.6	37.9
3. Pt (Rz)	12.6	0.8
4. Pt (Gn)	10.3	0.6
5. Pd/Pt (Rz) 1:1	84.6	4.6
6. Pd/Pt (Rz) 2:1	91.8	5.5
**7. Pd/Pt (Rz) 1:2**	**22.2**	0.3
8. Pd/Pt (Gn) 1:1	111.7	0.8
9. Pd/Pt (Gn) 2:1	182.1	1.3
**10. Pd/Pt (Gn) 1:2**	**54.1**	1.7

**Figure 2:**
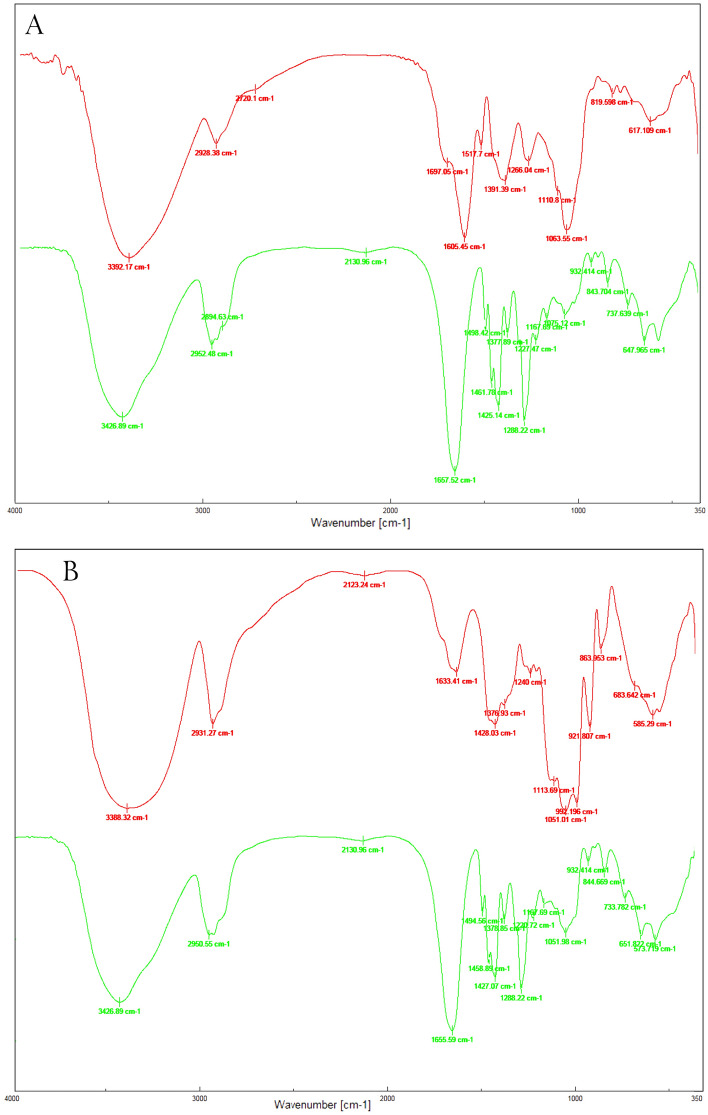
FT-IR spectra of Rosemary extract and 7 NPs (**A**), Ginseng extract and 10 NPs (**B**). In both pictures the upper spectrum is related to plant extract and the lower spectrum is NPs.

FT-IR analysis was exploited to investigate the various functional groups found in Rosemary and Ginseng extracts, along with their impact in the synthesis of nanoparticles prior to bio-reduction. To enhance our understanding of the procedure, NPs underwent centrifugation, washing with distilled water, drying, and subsequent FT-IR analysis. The Fourier-transform infrared spectroscopy conducted on plant extracts and nanoparticles revealed multiple peaks across different regions, illustrating the intricate composition of the biological materials involved in the reduction of metal ions^69^. The 3392, 2928, 2720, (1697, 1605), 1517, (wide peak in 1391), 1266, 1063, 819 and 617 cm^−1^ peaks of Rosemary extract were respectively moved to new positions 3427, 2952, 2894, a new peak in 2131, (1657), 1498, (1461, 1425, 1378), 1288, 1075, 843 and 647 in the region of 400–4000 cm^−1^ for **7** NPs (Fig. 2A). The FT-IR spectrum of Ginseng showed some peaks in 3388, 2931, 2123, 1633, (1440), 1428, 1376, 1240, 1113, 1051, 921, 864, 683 and 582 cm^−1^ that shifted to 3426, 2950, 2131, 1655, (1494, 1458), 1427, 1378, 1288, 1167, 1051, 932, 844, 651 and 573 cm^−1^ in **10** NPs (Fig. 2B).

**Figure 3:**
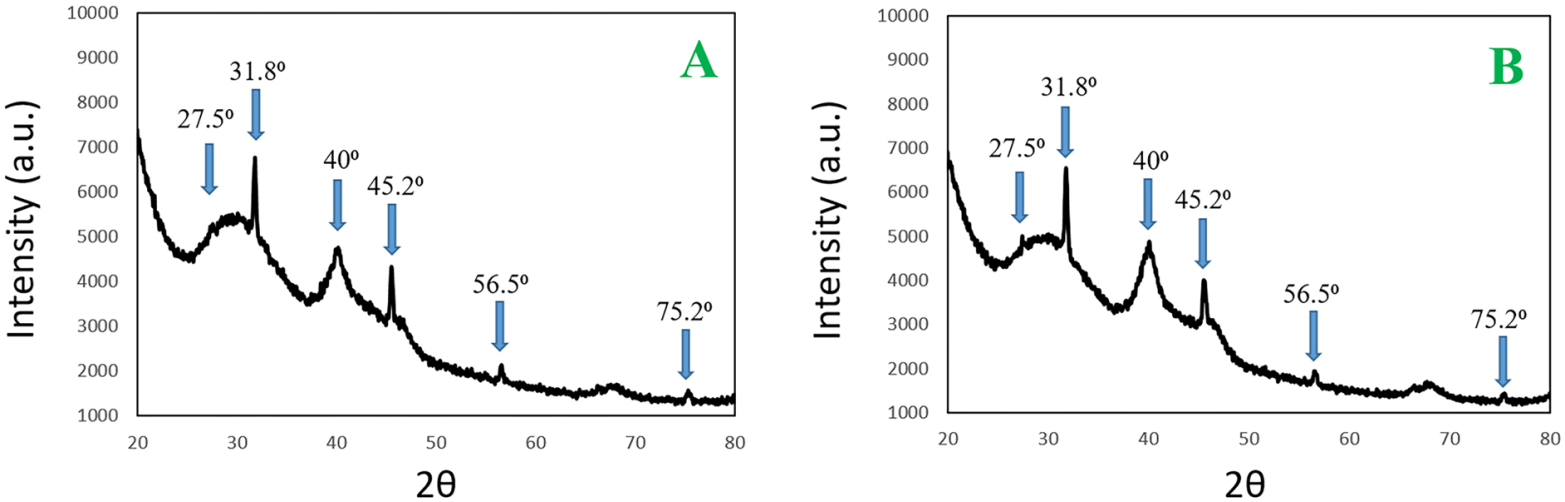
XRD patterns of 7 NPs (**A**) and 10 NPs (**B**).

Furthermore, a small amount of colloidal solutions from each sample was applied to a silicon substrate for the purpose of conducting XRD characterizations. The structure of the nanoparticles was examined in grazing incidence mode (with θ_inc_ = 0.1 Â°) using a rotating anode of Cu Kα radiation (λ = 1.540598 Å) operating at 45 kV and 200 mA. The XRD patterns of 7 NPs are shown in Fig. 3A, displaying six distinct diffraction peaks at 27.5°, 31.8°, 40°, 45.2°, 56.5° and 75.2°. Conversely, Fig. 3B shows the diffraction peaks for **10** NPs, which include similar angles of at 27.5°, 31.8°, 40°, 45.2°, 56.5° and 75.2°. These XRD peaks align with those of fcc Pt (JCPDF 04-0802) and Pd (JCPDF 46-1043). The mean size of the crystalline domain responsible for these diffraction patterns was predicted from the XRD spectra by the use of the Debye–Scherrer approximation (D = Kλ/βcosθ), resulting in dimensions of 67.53 ± 15.87 nm for **7** NPs and 98.32 ± 35.2 nm for **10** NPs^72,82^.

**Figure 4:**
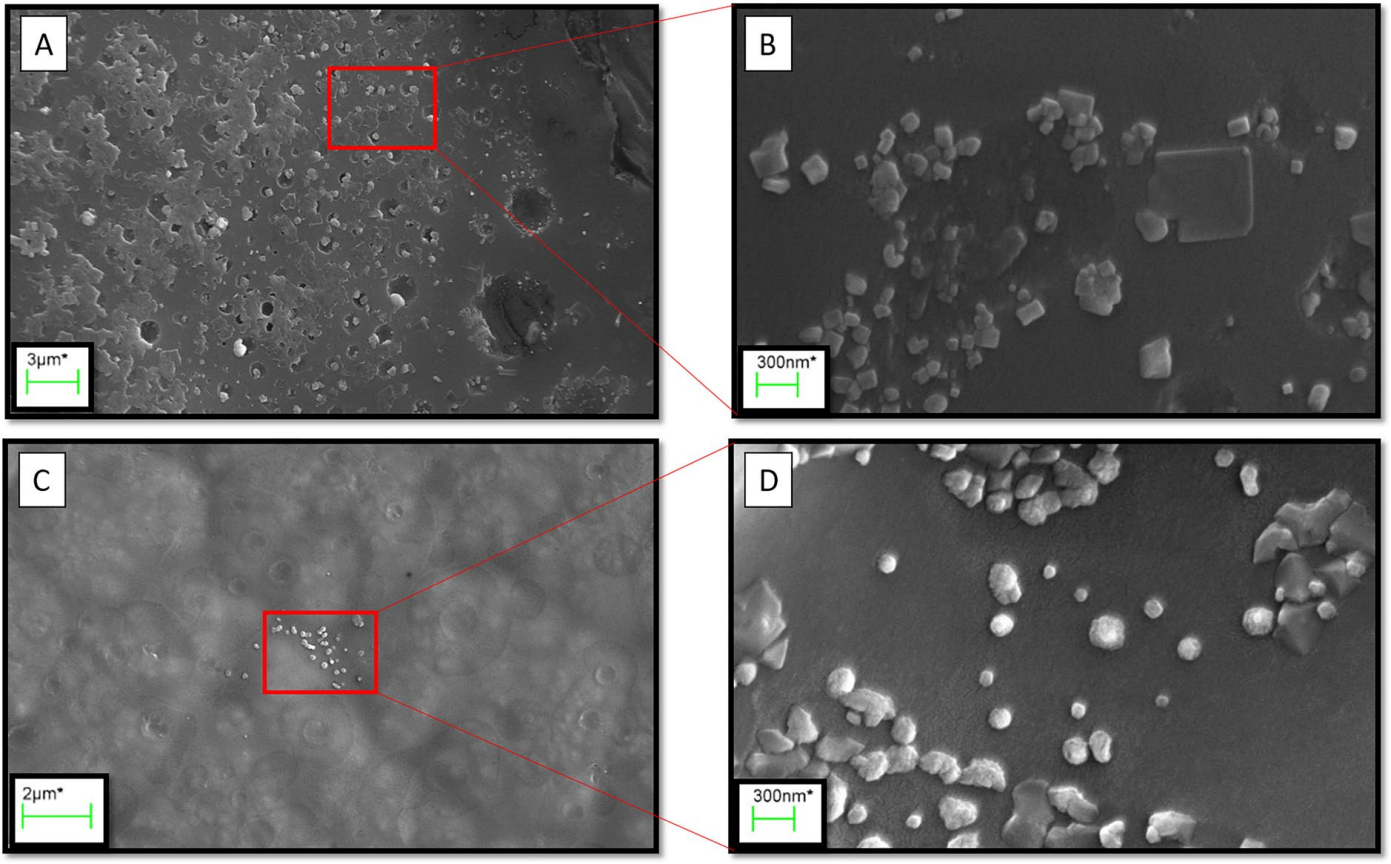
SEM image of nanoparticle 7 (**A**,**B**) and nanoparticle 10 (**C**,**D**).

**Figure 5:**
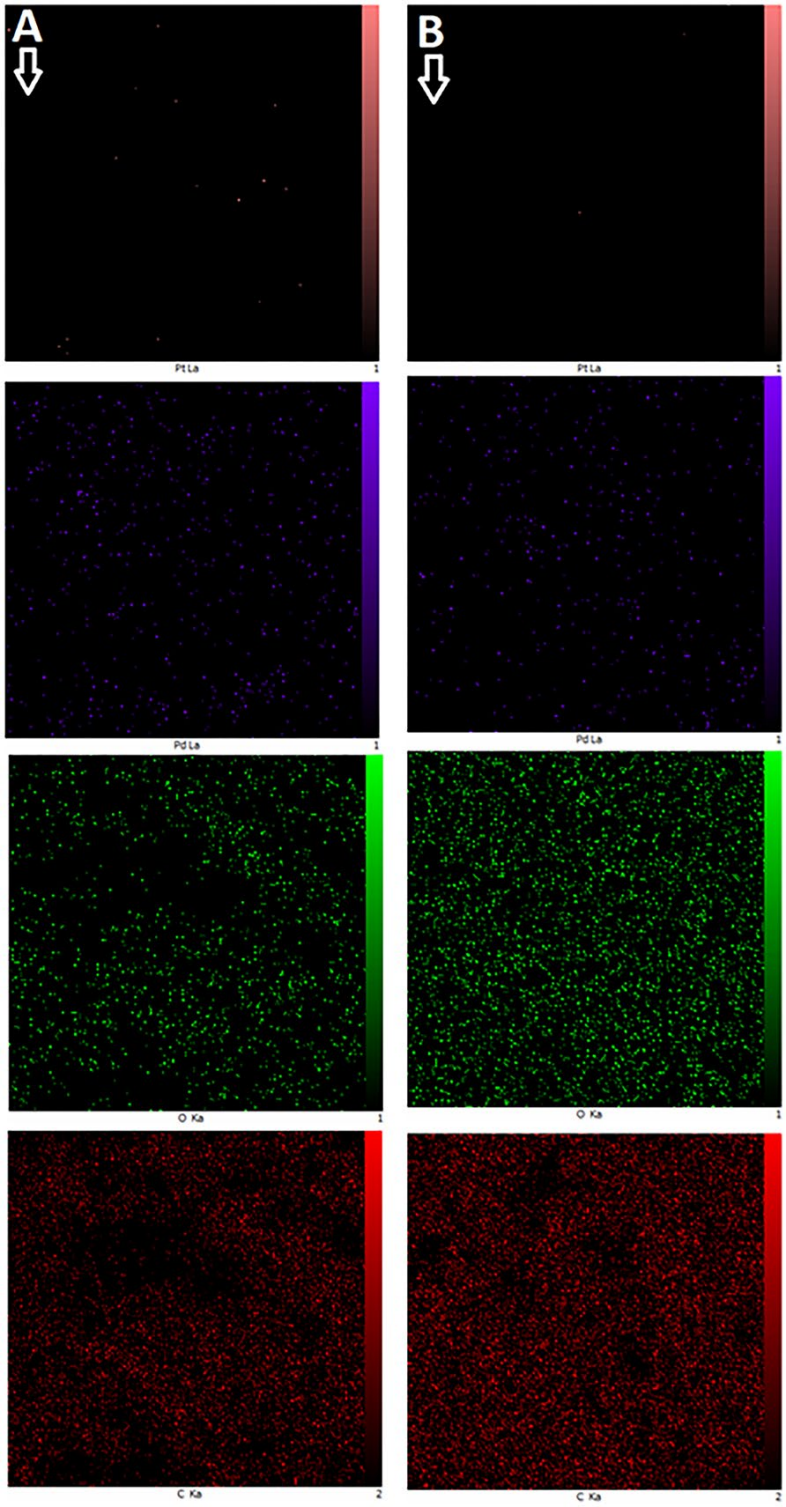
EDX elemental mapping of nanoparticle 7 (**A**) and nanoparticle 10 (**B**).

To conduct scanning electron microscopy (SEM), the nanoparticles were dispersed in water, and a drop of this suspension was deposited onto a carbon specimen sample holder. Figure 4 presents the SEM images of nanoparticles. In Fig. 4B, cubic nanoparticles with an average diameter of 94.12 ± 26.47 nm are shown, while Fig. 4D shows that the majority of particles have a spherical shape with an average particle size of 139.22 ± 53.99 nm. It is worth noting that the observed sizes are larger than those obtained through XRD patterns. The XRD sizes are attributed to crystalline sizes, while the SEM images may depict the size of several agglomerated crystals. Furthermore, the relevant EDX elemental mapping (Fig. 5) demonstrated a well-distributed composition of elements in the synthesized nanoparticles. For nanoparticle 7, the atomic percentages were as follows: 86.915, 10.995, 0.695, and 1.395 for C, O, Pd, and Pt, respectively. Correspondingly, for nanoparticle 10, the atomic percentages were 85.005, 13.485, 0.535, and 0.975, respectively. The results affirm the presence of the extract compounds in the nanoparticles compositions, thus corroborating the findings of the FT-IR analysis.

**Figure 6:**
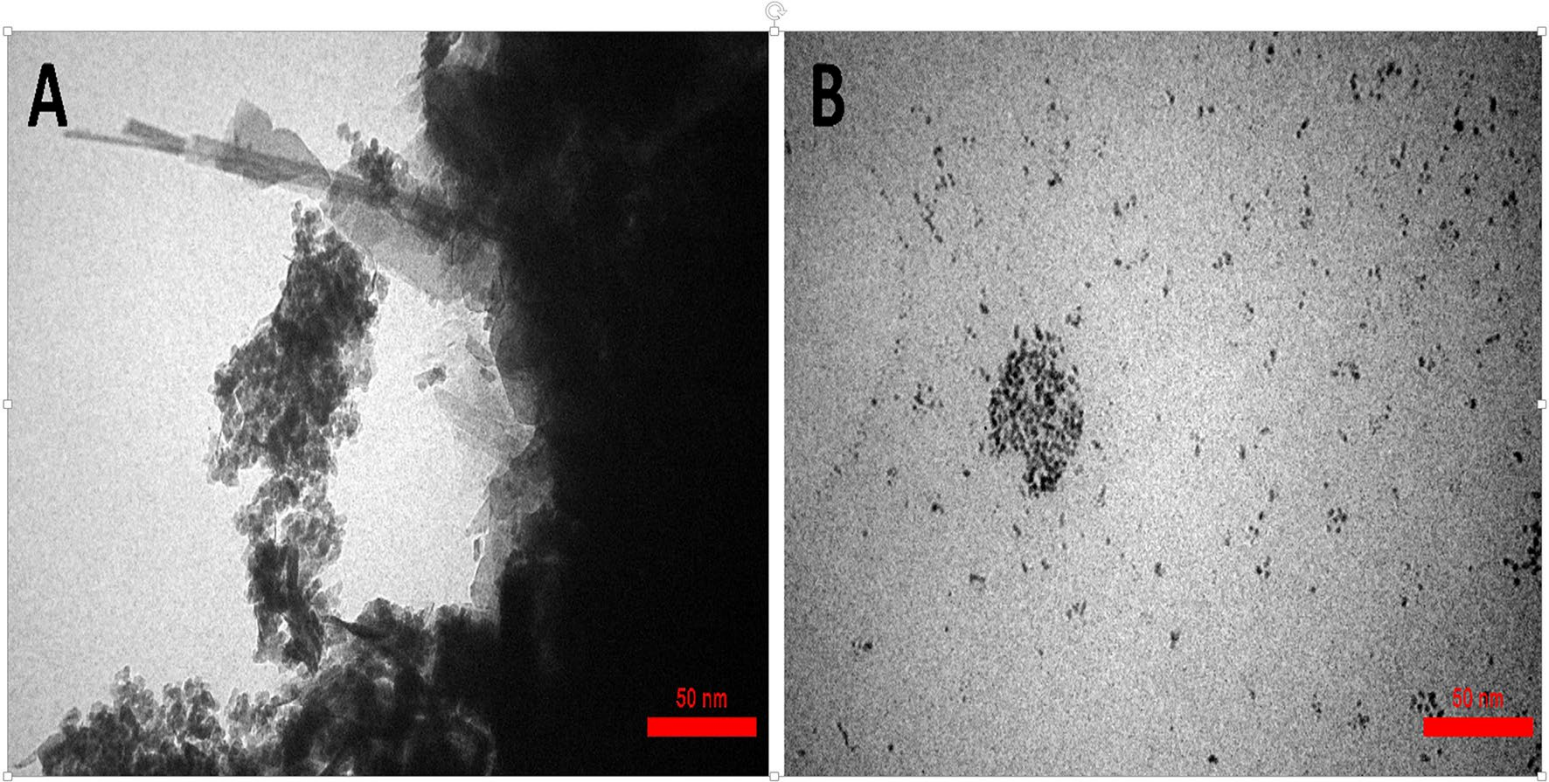
The TEM images of nanoparticle 7 (**A**) and nanoparticle 10 (**B**).

To further investigate the morphology of the nanoparticles, a droplet of the synthesized colloidal solutions for each sample was deposited onto a TEM grid and allowed to air-dry. Figure 6A is a TEM image of **7** NPs. This image reveals that the majority of particles exhibit diameters measuring approximately 3.7 ± 0.8 nm, although smaller and larger NPs are also present. The **10** NPs appear to be predominantly spherical, consistent with the TEM image depicted in Fig. 6. The distribution of diameters indicates a mean value of approximately 2.3 (± 0.1) nm.

### In vitro selective cancer cell cytotoxic activity

**Figure 7:**
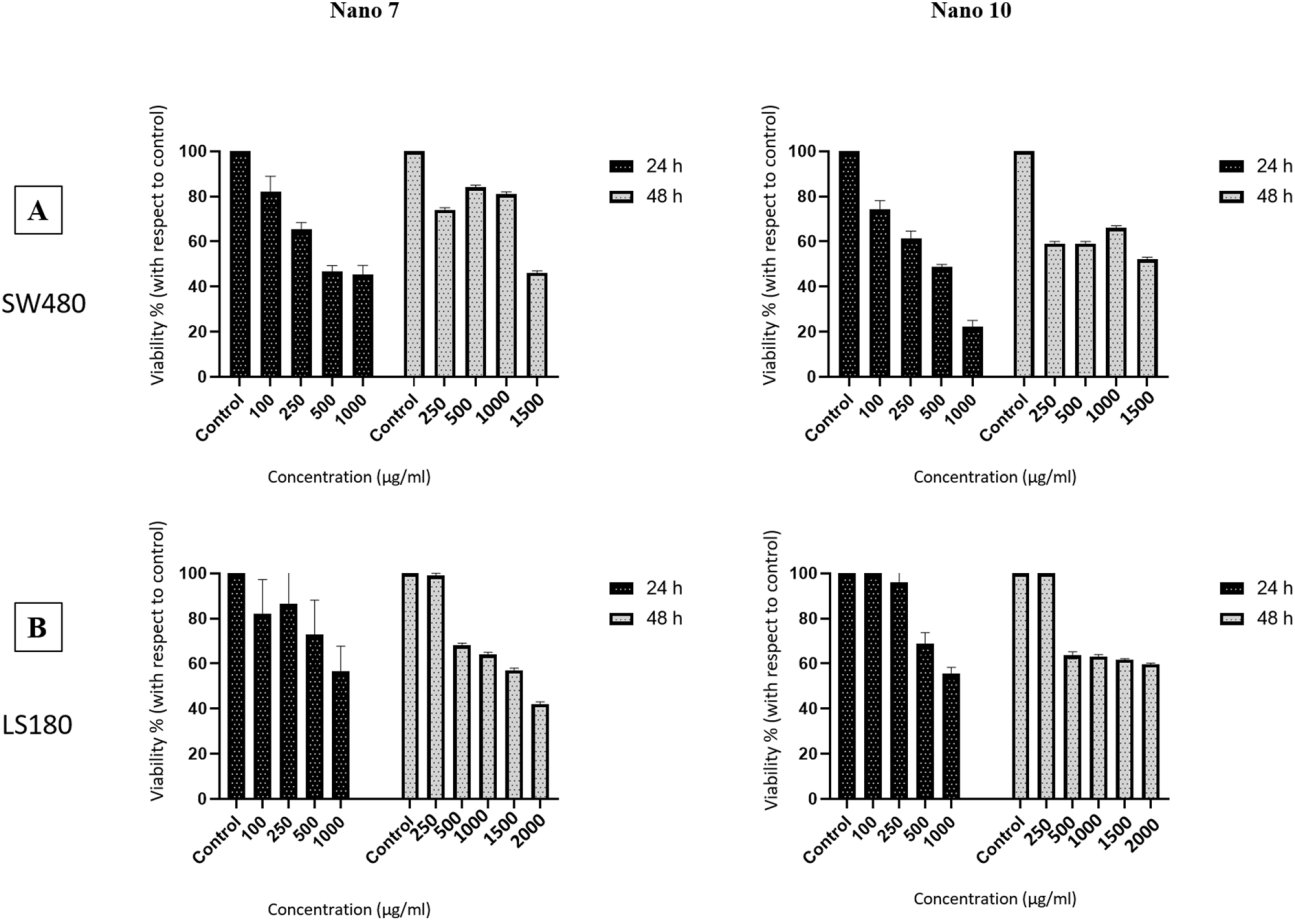
IC_50_ values for NPs 7 and 10, in cytotoxicity assay with colon cancer cell lines: SW480 (**A**) and LS180 (**B**).

The cytotoxic activity of the nanoparticles against SW480 and LS180 colorectal cancer cell lines was assessed through the MTT dye reduction method, a well-established bioassay used to measure the extent of an agent's specific detrimental effect on certain cells. Cytotoxicity, indicating the toxicity level towards cells, was evaluated by incubating cells with increasing nanoparticle concentrations for 24–48 h, followed by assessing anti-proliferative activity (see “Materials and methods” section). Table 3 and Fig. 7 provide the cytotoxicity data, represented as IC_50_ values, which correspond to the concentrations of a compound inhibiting 50% of cell growth. The results highlight that the degree of lethality and cytotoxicity of the synthesized nanoparticles are influenced by the type of plant extract employed in their synthesis. Moreover as can be seen in Fig. 7, different concentrations of nanoparticles solution were treated on the cell lines and compared with the control. The results showed that the presence of nanoparticles in the culture medium prevented cell survival and growth, and compared to the control culture medium that did not receive any drug, the cytotoxicity rate increased in the treatment culture mediums with increasing concentration. Many studies have investigated the effect of metal nanoparticles, especially palladium and platinum nanoparticles, on colon cancer cell lines. For example, in a research, it was determined that the effective dose of a bimetallic palladium-platinum nanoparticle on the SW480 cell line is 50 µg/mL^83^. In addition, in another study, the effect of 75 µg/mL of palladium-platinum bimetallic nanoparticles on the same cell line has been reported^84^. It should be noted that the concentration values reported in these studies are lower than the values obtained in our research, but the current research is acceptable to a large extent due to the fact that an environmentally friendly synthesis method was used for the synthesis of nanoparticles. It seems that the method of synthesis and the used solvent have a great effect on the purity and anti-cancer property of the nanoparticle. Furthermore the effective doses indicate that the achieved concentrations are notably more efficient than those reported in previous studies involving plant extracts alone on colorectal cell lines. The results of rosemary extract on SW480 cell line have shown that the IC_50_ value is 500 µg/mL^48^. Consequently, it is evident that nanoparticles outperform the plant extracts^48,85^.

**Table 3:** Selective cytotoxicity data, stated as IC_50_, of the compounds opposing cancer cells.

Nanoparticles	IC_50_ (µg/mL) ± SD			
	SW480		LS180	
	24 h	48 h	24 h	48 h
**7**	436.3 ± 0.2	1290 ± 04	790 ± 0.45	1221 ± 0.21
**10**	320.6 ± 0.06	712 ± 0.1	946 ± 0.87	1613 ± 0.9

Research on the function and mechanism of different antitumor drugs such as 5 fluorouracil (5-FU), mitomycin (MMC), cisplatin (CP), all-trans retinoic acid (ATRA), TRAIL, doxorubicin (DOX) and oxaliplatin (OP) has been done on the SW480 cell line^86–89^. For example, the IC_50_ values reported for oxaliplatin and DOX on this cell line are 2.1 ± 0.3 and 65.25 ± 3.48 µM, respectively^88,90^. Also, the effect of known antitumor drugs on LS180 cell line has been investigated^91^. For instance, the IC_50_ values for cisplatin, oxaliplatin and carboplatin on LS180 cell lines are 4.1 ± 0.1, 1.7 ± 0.8 and 147.1 ± 19.4 μM, respectively^92^. Although these drugs have excellent efficacy, due to the side effects and drug resistance they cause, scientists have recently paid attention and focus to the use of drugs with less damage.

### Nanoparticles effect on the expression levels of autophagy-related genes

Researchers have illuminated a set of mechanisms in the toxicity of nanoparticles such as apoptosis, necrosis, oxidative stress and autophagy. Among the mechanisms, autophagy was recently recognized as an important style of cell death in various toxicity caused by nanoparticles. Macroautophagy consists of three steps: cytoplasmic constituents (mis-folded proteins or damaged organelles) are enveloped by double-membraned autophagosome; autophagosomes are fused with lysosome to form an autolysosome; and the cytoplasmic constituents are degraded and recycled in lysosome. Autophagy is likened to the opposite faces of Janus, as it could protect cells to survive under certain severe conditions, as well as inducing cell death when too much autophagy occurs. Similarly, nanoparticles-mediated autophagy during nanotoxicity might be an adaptive cellular response aiding in the clearance of nanoparticles, and also might be harmful in cellular dysfunction^93^.

Research conducted in recent years indicate that various metal and metal oxide-based nanoparticles can show good anti-cancer properties by affecting the autophagy process^94^. For example, silver nanoparticles reduced the viability of PANC-1 pancreatic adenocarcinoma cells and induced apoptotic and autophagic cell death significantly more than non-tumor cells. In addition, the level of autophagy marker protein LC3-II was significantly increased in PANC-1 cells treated with silver nanoparticles, thus indicating that apoptotic and necroptosis cell death occurs with autophagy^95^. In recent research, it was found that silver nanoparticles affect the function of lysosome and autophagic flux by reducing the expression of TFEB in A459 lung cancer cells and lead to cell damage^96^. In another research, it was found that gold nanoparticles cause autophagy at the same time as oxidative stress on human lung cancer cell line. Also, the formation of autophagosomes along with the absorption of nanoparticles in lung fibroblasts and the positive regulation of autophagy proteins, microtubule-associated protein 1 light chain 3 (MAP-LC3) and autophagy gene 7 (ATG7) were observed in the treated samples^97^. Some researchers have developed various forms of autophagy-targeted nanodrug delivery systems to treat tumors, leading to advances in cancer therapy. The mechanism of autophagy induction by nanomaterials is thought to be mainly mediated by intracellular oxidative stress^98^.

**Figure 8:**
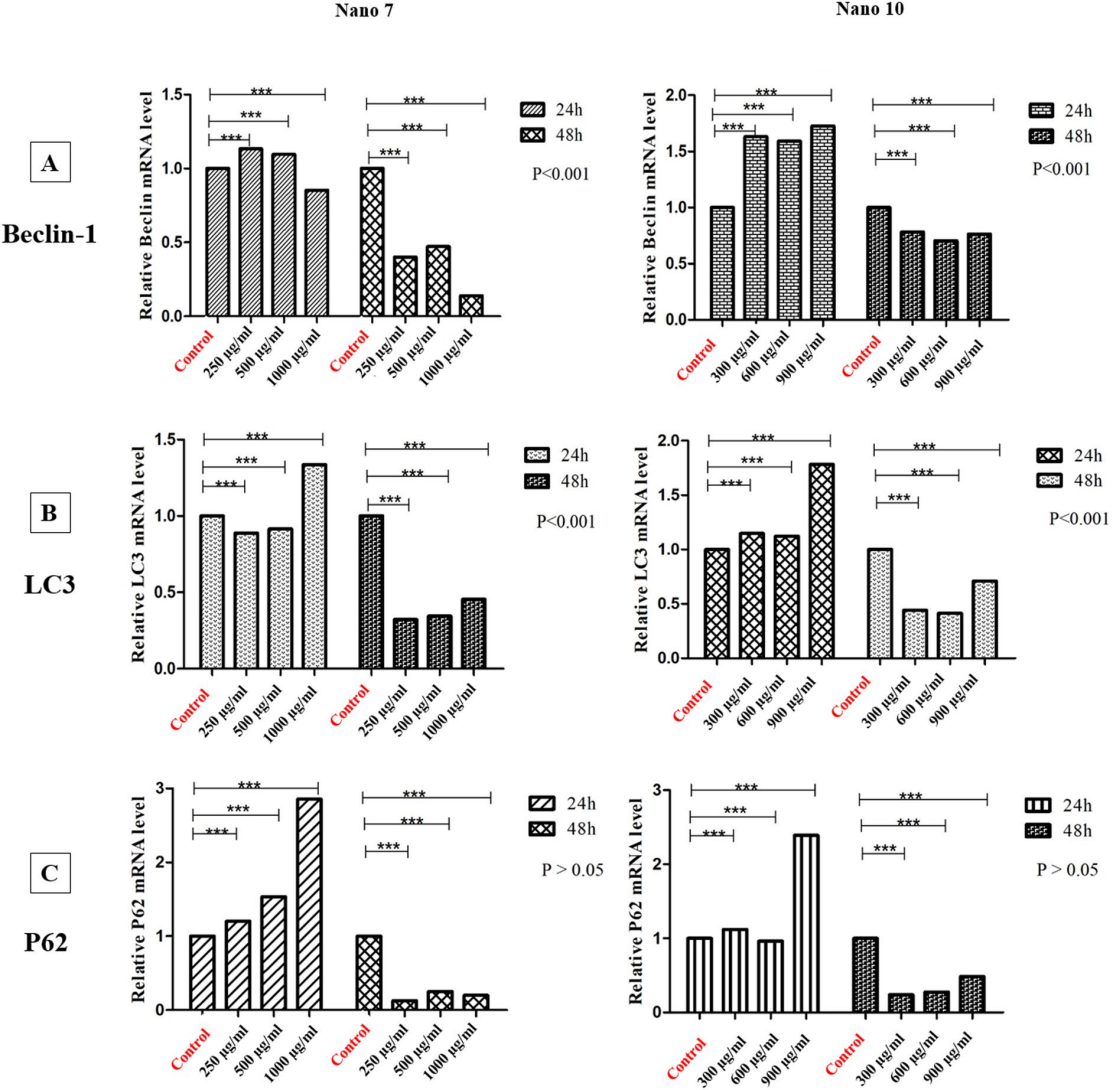
Comparison of the relative mRNA expression levels of Belin-1 (**A**), LC3 (**B**), and P62 (**C**) of LS180 cells. The mRNA expression levels of the corresponding genes were determined by q RT-PCR and normalized to GAPDH expression levels in 24 and 48 h for 7 NPs and 10 NPs.

The studies revealed that both nanoparticles have a significant impact on the expression of autophagy genes, with varying results based on the specific gene, the duration and dosage of the nanoparticles, and the nature of the cell line. As shown in Fig. 8, when examining the effect of nanoparticle 7 on the LS180 cell line, it becomes apparent that the Beclin-1 gene experiences an increase after 24 h at concentrations of 250 and 500 µg/mL. This suggests the initiation of the autophagy process, and after 48 h, autophagy is inhibited at all concentrations. So it can be said that this process may be dependent on time and dosage, having a prolonged impact on cancer cells On the other hand, the LC3 gene shows an increase only at the concentration of 1000 µg/mL. This observation indicates autophagosome accumulation within 24 h, followed by autophagy flux inhibition at higher concentrations, eventually leading to cellular toxicity and apoptosis. In other words, lower concentrations induce autophagy initiation, while higher concentrations result in toxicity. Over the 48-h period, autophagy inhibition occurs initially. Consequently, if we aim for a rapid impact, lower concentrations are preferable, while for longer-term applications, the difference in concentration is less crucial due to early autophagy inhibition. The behavior of the P62 gene resembles that of LC3, with the most pronounced effect observed at the 1000 µg/mL concentration after 24 h, followed by the early-stage inhibition of autophagy after 48 h.

Regarding nanoparticle 10, akin to nanoparticle 7, there is an increase in autophagy activity within the initial 24 h. In a shorter timeframe, the autophagy flux is inhibited, eventually leading to the final stage of autophagy being blocked. Conversely, over a more extended period, the initial phase of autophagy is inhibited. Overall, in all experiments, autophagy flux inhibition was consistently observed at the highest concentration within the initial 24-h interval (see Fig. 8).

**Figure 9:**
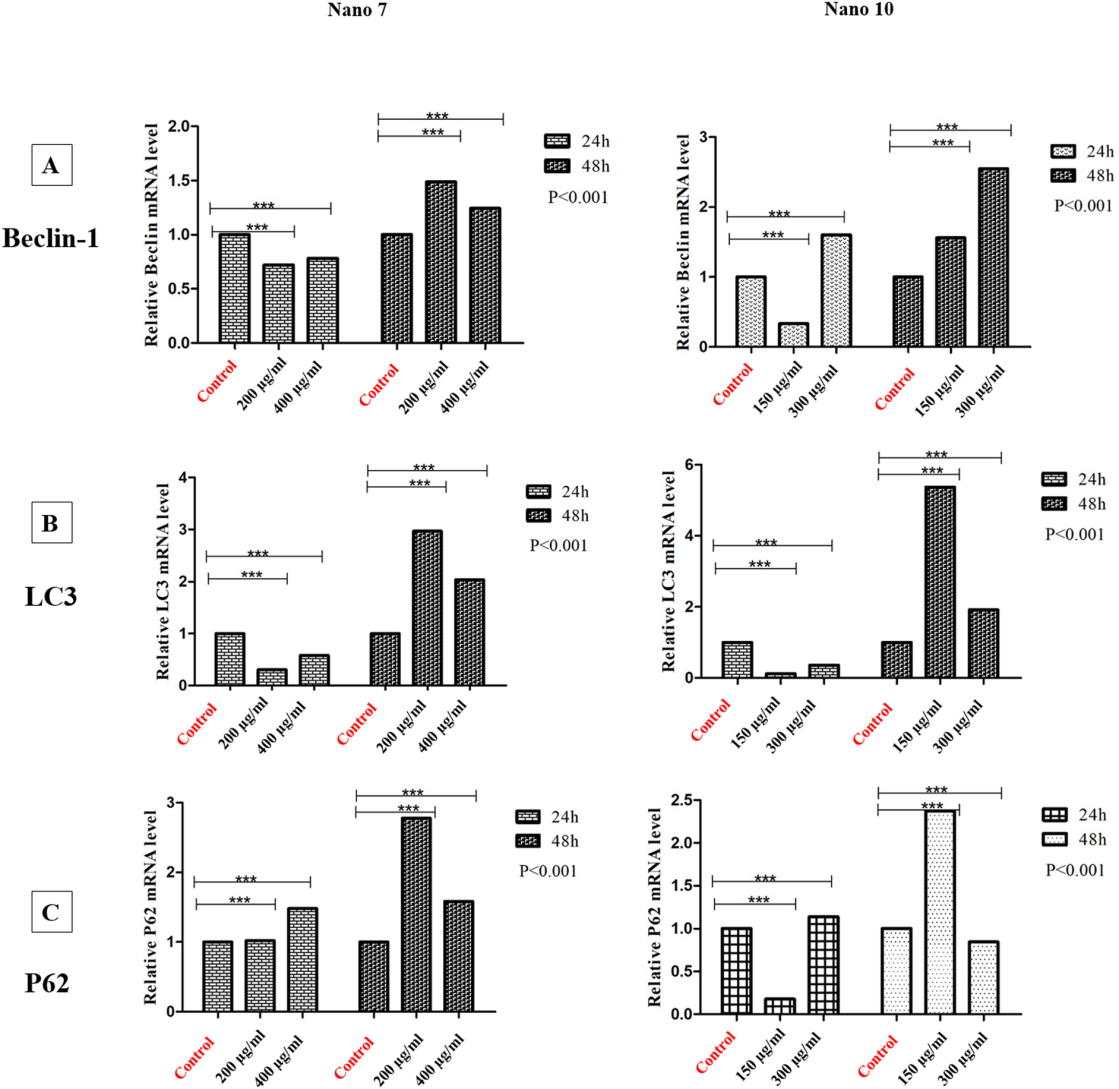
Comparison of the relative mRNA expression levels of Belin-1 (**A**), LC3 (**B**), and P62 (**C**) of SW480 cells. The mRNA expression levels of the corresponding genes were determined by q RT-PCR and normalized to GAPDH expression levels in 24 and 48 h for 7 NPs and 10 NPs.

When it comes to the SW480 cell line, the results are different. Examining the effect of nanoparticle 7 on Beclin-1 and LC3 genes shows a decrease in gene expression in a shorter timeframe, followed by an increase over a more extended period. Notably, the P62 gene demonstrated more pronounced effects at 48 h compared to 24 h, with a general lack of dose dependence. To elaborate, the initial stage of autophagy was impacted at 24 h, while the autophagy flux was affected at 48 h (see Fig. 9). Nanoparticle 10 was also investigated on the SW480 cell line. Beclin-1 results showed that it inhibited autophagy at low concentrations, but stimulated the initial stages of autophagy at a concentration of 300 µg/mL, similarly occurring over 48 h. As for LC3 and P62 genes, low concentrations of nanoparticles within 24 h hindered the initial stage of autophagy, with the 150 µg/mL concentration proving to be notably effective. But in 48 h, both concentrations inhibited the autophagy flux (see Fig. 9).

The SW480 cell line has c-myc, Kras, and p53 oncogenes. Kras and c-myc mutation lead to autophagy activity, and P53, a key player in autophagy, is compromised in this cell line. Considering the influence of these oncogenes on autophagy, it is highly likely that they disrupt the normal and logical processes in this cell line.

## Conclusions

Present paper employed an environment-friendly approach to synthesize palladium and platinum nanoparticles. For this purpose, the synthesis utilized extracts from rosemary and ginseng plants, both known for their anticancer properties. Then, the size and structure of these nanoparticles were scrutinized through the use of conventional methods, like DLS analysis, FT-IR spectroscopy, XRD, SEM, EDX and TEM techniques, revealing their nanoscale dimensions suitable for biological applications. Two bimetallic nanoparticles (7 and 10NPs) were chosen for further investigation and evaluation with cell cultures. The findings indicate a significant impact of both nanoparticles on the tested cancer cell lines. The results showed that the nanoparticles synthesized by an environmentally friendly method with rosemary and ginseng extract had an acceptable effect on colon cancer cell lines (SW480 and LS180), which had a better effect compared to the plant extract alone. Furthermore, the study explored the influence of nanoparticles on the expression of autophagy-related genes, yielding diverse outcomes based on nanoparticle concentration, experiment duration, gene type, and cell line characteristics.

## Data Availability

All datasets analyzed during the current research are available from the corresponding author on reasonable request.
